# Anti-Inflammatory Effects of *Apostichopus japonicus* Extract in *Porphyromonas gingivalis*-Stimulated RAW 264.7 Cells

**DOI:** 10.3390/cimb46120799

**Published:** 2024-11-24

**Authors:** Min-jeong Kim, Hyun-jin Kim

**Affiliations:** 1 Department of Convergence Technology for Food Industry, Wonkwang University, Iksan 54538, Republic of Korea; lucklove519@naver.com; 2 Institute of Biomaterial • Implant, Department of Oral Anatomy, School of Dentistry, Wonkwang University, Iksan 54538, Republic of Korea

**Keywords:** *Apostichopus japonicus*, anti-inflammatory, *Porphyromonas gingivalis*, periodontitis, NF-κB, MAPK, RAW 264.7 cells

## Abstract

*Apostichopus japonicus* has been used both as a food and in traditional medicine. However, its anti-inflammatory effects in periodontal diseases have not been studied. We examined the anti-inflammatory properties of *Apostichopus japonicus* extract in RAW 264.7 cells stimulated by *Porphyromonas gingivalis*. The cytotoxicity of *Apostichopus japonicus* extract was evaluated using the MTS assay. Its effect on NO production was then measured using the NO assay. The mRNA expression of inducible nitric oxide synthase (iNOS) and the pro-inflammatory cytokines IL-1β and IL-6 were assessed using quantitative real-time PCR (qRT-PCR). Western blotting was performed to investigate the expression of regulatory proteins involved in the NF-κB and MAPK signaling pathways. *Apostichopus japonicus* extract significantly inhibited NO production without cytotoxicity in RAW 264.7 cells. Following *Porphyromonas gingivalis* stimulation, treatment with the extract decreased iNOS mRNA expression and protein levels, which are responsible for NO production. The extract also suppressed the mRNA expression of pro-inflammatory cytokines. Additionally, *Apostichopus japonicus* extract inhibited NF-κB activation by regulating signaling molecules such as IKK and IκBα, while also preventing the phosphorylation of MAPK, including ERK, p38, and JNK, showing anti-inflammatory potential. Therefore, it may be a promising natural candidate for the development of new preventive and therapeutic strategies for periodontitis.

## 1. Introduction

The inflammatory response is a natural reaction of the body to tissue damage caused by physical injuries, harmful chemicals, or the presence of pathogens such as bacteria and viruses [1,2]. This response is essential for initiating the healing process because it helps contain damage, eliminate harmful agents, and promote tissue repair. When tissue damage occurs, various immune cells, including macrophages, neutrophils, and lymphocytes, are activated and migrate to the affected area [3]. These cells play a crucial role in detecting and removing harmful agents. They also release signaling molecules known as cytokines and chemokines, which coordinate the actions of other immune cells, amplifying the response [4,5].

Macrophages are key immune cells that help protect the host from damage and infection. They play an important role in the inflammatory response triggered by lipopolysaccharide (LPS), a component of the outer membrane of Gram-negative bacteria [6,7]. LPS binds to Toll-like receptor 4 on the surface of macrophages, activating the transcription factor nuclear factor kappa B (NF-κB) through the mitogen-activated protein kinase (MAPK) pathway, which includes p38 and c-Jun N-terminal kinase (JNK), as well as extracellular signal-regulated kinase (ERK) [8]. This process leads to the expression of enzymes such as inducible nitric oxide synthase (iNOS), which induces the production of inflammatory mediators such as nitric oxide (NO). Additionally, numerous inflammatory mediators amplify the inflammatory response by inducing the release of pro-inflammatory cytokines including interleukin (IL)-1β and IL-6 [9,10,11]. Therefore, inhibiting the activation of NF-κB and MAPKs and reducing the production of inflammatory mediators and pro-inflammatory cytokines is considered a key therapeutic strategy for evaluating anti-inflammatory effects.

Periodontitis is a chronic inflammatory disease resulting from persistent bacterial infection in the tissues that support and surround the teeth. This condition is primarily driven by the accumulation of bacterial plaque on the tooth surface, leading to a prolonged immune response that damages both the soft and hard tissues of the teeth [12,13,14]. The primary pathogen involved in periodontitis is *Porphyromonas gingivalis* (*P. gingivalis*), a Gram-negative anaerobic bacterium. This bacterium is particularly virulent as it produces powerful proteases known as gingipains, which degrade host proteins and facilitate bacterial invasion [13,15]. Gingipains not only contribute to tissue destruction but also directly interact with the host’s immune system, activating immune cells such as macrophages and dendritic cells within periodontal tissues, stimulating the release of various pro-inflammatory cytokines including tumor necrosis factor-alpha (TNF-α), IL-1β, and IL-6 [16,17]. These cytokines play a critical role in regulating the inflammatory response. As *P. gingivalis* infection progresses, this prolonged immune–inflammatory response causes gradual damage to periodontal tissues, which can result in tooth loss if left untreated.

Antimicrobials such as cetylpyridinium chloride, chlorhexidine, and triclosan have been used to treat periodontitis. However, their use is associated with certain limitations, including side effects such as dry mouth and the potential for antibiotic resistance with long-term use [18,19]. Additionally, current treatments for periodontitis demonstrate limited efficacy and offer minimal preventive benefits, highlighting the need for new therapeutic developments to address these challenges. Moreover, periodontitis is linked to various chronic conditions, including Alzheimer’s disease, cardiovascular diseases, stroke, and diabetes [20,21], underscoring the critical role of oral health in overall health management. In this context, there is a growing demand for the discovery of safer therapeutic agents with selective anti-inflammatory effects against periodontitis-associated bacteria.

*Apostichopus*, classified under the class Holothuroidea within the phylum Echinodermata, are a diverse group of marine invertebrates, with approximately 1500 species distributed globally. These organisms are notable not only for their ecological role in marine ecosystems but also as a food source and have been used in traditional medicine in various cultures [22,23]. They are particularly recognized for their potential health benefits, including aiding in fatigue recovery and improving kidney function, with studies indicating that they are relatively safe for these purposes [22,24]. Previous studies on *Apostichopus japonicus* (*A. japonicus*), one of the *Apostichopus* species, have reported beneficial properties, including antioxidant and anti-allergic activities, in LPS-stimulated macrophages [25,26,27]. These findings suggest that compounds derived from *A. japonicus* may play a significant role in modulating immune responses and protecting against oxidative stress.

Despite these promising characteristics, the anti-inflammatory effects of *A. japonicus* on periodontitis have not been reported. Therefore, this study aimed to investigate the anti-inflammatory signaling mechanisms of *A. japonicus *extract against periodontitis in vitro. The findings, which highlight the beneficial properties of *A. japonicus*, are expected to provide new therapeutic approaches for oral health.

## 2. Materials and Methods

### 2.1. Chemicals and Reagents

The CellTiter 96 AQueous One Solution Cell Proliferation Assay and Griess Reagent System were purchased from Promega (Madison, WI, USA). *IL-1β*, *IL-6*, *iNOS*, and *β-actin* oligonucleotide primers were purchased from Bioneer (Daejeon, South Korea). Antibodies targeting iNOS, phosphorylated Erk1/2 (p-Erk1/2), Erk1/2, phosphorylated p38 (p-p38), p38, phosphorylated JNK (p-JNK), JNK, phosphorylated IκB kinase (IKK)α/β (p-IKKα/β), phosphorylated NF-κB inhibitor α (IκBα) (p-IκBα), IκBα, and NF-κB (p65 subunit), as well as horseradish peroxidase (HRP)-linked anti-rabbit IgG, were purchased from Cell Signaling Technologies (Danvers, MA, USA). Antibodies targeting β-actin were purchased from Sigma-Aldrich (St. Louis, MO, USA). Antibodies targeting proliferating cell nuclear antigen (PCNA) and m-IgGκ were purchased from Santa Cruz Biotechnology (Dallas, TX, USA). Heat-killed *P. gingivalis* was provided by OraTix Inc. (Seoul, Republic of Korea).

### 2.2. A. japonicus Extract

The *A. japonicus* extract used in this study was kindly provided by Professor Han-Kil Choi’s laboratory in the Department of Life Sciences at Wonkwang University (Iksan, Republic of Korea). Specimens were purchased from Taean Geunheung-myeon fisheries cooperatives (Taean, Republic of Korea) and transported to the laboratory under refrigerated conditions. After thorough washing with tap water and cutting into 0.5–1 cm lengths, the specimens were extracted using absolute ethanol (99%) at a ratio of 10 times the weight of the sample. The extraction process was performed over three cycles, each lasting 3 days, to maximize the yield of bioactive compounds. The extract was then purified, dialyzed to remove salts, and freeze-dried. The freeze-dried extract was reconstituted with dimethyl sulfoxide to the indicated concentrations and stored at −20 °C until further use.

### 2.3. High-Performance Liquid Chromatography (HPLC) Analysis

The HPLC analysis of *A. japonicus* extract was conducted using a Waters 2690 XE separations module (Waters, Milford, MA, USA). Chromatographic separation was achieved using a Waters SunFire C18 column (3.5 μm, 4.6 mm × 150 mm) set at 35 °C. A 10 μL sample was injected and eluted at a flow rate of 1 mL/min. The mobile phase system consisted of 0.1% formic acid in distilled water (buffer A) and 0.1% formic acid in acetonitrile (buffer B). The elution conditions were programmed as follows: initial conditions from 0.0 to 1.0 min, 95% A and 5% B; from 1.0 to 12.0 min, maintenance at 95% A and 5% B; from 12.0 to 14.0 min, a gradient from 95% to 0% A and 5% to 100% B; from 14.0 to 16.0 min, 0% A and 100% B were maintained; from 16.0 to 20.0 min, a gradient from 0% to 95% A and 100% to 5% B; and from 20.0 min onward, maintenance at 95% A and 5% B. The sampling was run for a total of 20 min, and UV monitoring was performed at 205 nm.

### 2.4. Cell Culture

Murine macrophage RAW 264.7 cells (ATCC, Rockville, MD, USA) were cultured in Dulbecco’s modified Eagle medium (Gibco BRL, Carlsbad, CA, USA) supplemented with 10% fetal bovine serum (Gibco BRL) and 1% antibiotic–antimycotic solution (Gibco BRL). Cells were maintained at 37 °C in a humidified atmosphere of 95% air and 5% CO_2_.

### 2.5. Cell Viability Assay

Cell viability was evaluated using the 3-(4,5-dimethylthiazol-2-yl)-5-(3-carboxymethoxy-phenyl)-2-(4-sulfophenyl)-2H-tetrazolium (MTS) assay, a colorimetric method that measures metabolic activity. This assay was performed following the manufacturer’s protocol (CellTiter 96® AQueous One Solution Cell Proliferation Assay, Promega, Madison, WI, USA), which relies on the reduction of MTS tetrazolium compound to formazan by the mitochondrial enzymes in viable cells. RAW 264.7 cells were seeded at a density of 1 × 10⁵ cells/well in a 96-well plate and cultured overnight in a 5% CO_2_ incubator (Thermo Fisher Scientific, Marietta, OH, USA). Subsequently, the cells were treated with *A. japonicus* extract at concentrations ranging from 0.0125 to 0.2 mg/mL for 24 h. The absorbance was measured at 490 nm using a microplate reader (TECAN, Mänedorf, Switzerland).

### 2.6. NO Assay

The NO assay was conducted using the Griess reaction, a well-established method for detecting nitrite, a stable metabolite of nitric oxide. The procedure was performed as outlined by Green et al. [28], with modifications made as necessary to suit the experimental conditions. RAW 264.7 cells were seeded at a density of 5 × 10⁵ cells/well in a 24-well plate and incubated overnight in a 5% CO_2_ incubator (Thermo Fisher Scientific). Subsequently, the cells were pretreated with various concentrations of *A. japonicus* extract (0.0125, 0.025, 0.05, and 0.1 mg/mL) for 2 h, following which the cells were stimulated with 1 × 10^7^ colony forming units (CFU)/mL of *P. gingivalis* for 24 h. Subsequently, cell culture supernatants were mixed with Griess reagent and incubated at room temperature for 10 min. The absorbance was measured at 540 nm using a microplate reader. The concentration of NO was calculated using a NaNO_2_ standard curve.

### 2.7. Quantitative Real-Time PCR (qRT-PCR)

RAW 264.7 cells were seeded at a density of 5 × 10⁵ cells/well in a 24-well plate and cultured overnight. Subsequently, the cells were pretreated with various concentrations of *A. japonicus* extract for 2 h and then stimulated with *P. gingivalis* (1 × 10^7^ CFU/mL) for 24 h. Total RNA was isolated using TRIzol reagent (Ambion, Carlsbad, CA, USA), and its concentration and purity were measured at A260/A280 using a Biospec-Nanodrop (Shimadzu, Nakagyo-ku, Kyoto, Japan). Total RNA was reverse-transcribed into cDNA using the PrimeScript RT Reagent kit (TaKaRa, Shiga, Japan). The cDNA was mixed with the PowerSYBR Green PCR Master Mix (Applied Biosystems, Warrington, UK) and specific primers for IL-1β, IL-6, iNOS, and β-actin. qRT-PCR was performed using the StepOnePlus Real-Time PCR System (Applied Biosystems, Foster City, CA, USA). The qRT-PCR cycling conditions were as follows: 95 °C for 10 min, followed by 40 cycles of 95 °C for 15 s and 60 °C for 1 min. Cycle threshold values were calculated using generated PCR curves, and *β-actin* mRNA expression was used as a loading control to normalize the expression of *IL-1β*, *IL-6*, and *iNOS* mRNA. The following primers were used for qRT-PCR—*IL-1β*: forward 5′-GAAAGACGGCACACCCACCCT-3′ and reverse 5′-GCTCTGCTTGTGAGGTGCTGATGTA-3′ (NM_008361); *IL-6*: forward 5′-GATGGATGCTACCAAACTGGA-3′ and reverse 5′-TCTGAAGGACTCTGGCTTTG-3′ (NM_031168); *iNOS*: forward 5′-AAGTCAAATCCTACCAAAGTGA-3′ and reverse 5′-CCATAATACTGGTTGATGAACT-3′ (NM_010927); and *β-actin*: forward 5′-CATCACTATTGGCAACGAGC-3′ and reverse 5′-GACAGCACTGTGTTGGCATA-3′ (NM_007393).

### 2.8. Western Blotting Analysis

RAW 264.7 cells were seeded at a density of 5 × 10^6^ cells/dish in 60 mm dishes and cultured overnight. Subsequently, the cells were pretreated with various concentrations of *A. japonicus* extract for 2 h and then stimulated with *P. gingivalis* (1 × 10^7^ CFU/mL) for the indicated time. The cells were then collected and washed twice with cold phosphate-buffered saline. Cytoplasmic and nuclear fractions were separated using a nuclear extraction kit (Cayman, Ann Arbor, MI, USA) according to the manufacturer’s instructions. The protein concentration was measured using a Pierce BCA Protein Assay Kit (Thermo Fisher Scientific, Rockford, IL, USA). Each sample was loaded onto a 10% sodium dodecyl sulfate-polyacrylamide gel and electrophoretically separated. The proteins were then transferred to polyvinylidene fluoride membranes. The membrane was blocked with 5% skim milk in Tris-buffered saline with Tween 20 (TBST) for 1 h at room temperature and incubated overnight at 4 °C with primary antibodies. The primary antibody dilutions used were as follows: iNOS (1:1000), NF-κB p65 subunit (1:1000), p-IκBα (1:1000), IκBα (1:1000), p-IKKα/β (1:1000), p-Erk1/2 (1:1000), Erk1/2 (1:1000), p-p38 (1:1000), p38 (1:1000), p-JNK (1:1000), JNK (1:1000), β-actin (1:5000), and PCNA (1:1000). After washing with TBST, the membranes were incubated with HRP-conjugated secondary antibodies for 1 h at room temperature. Protein bands were then detected using a chemiluminescent HRP substrate reagent (Millipore, Billerica, MA, USA) and cSeries Capture Software version 2.1.4.0731 (Azure Biosystems, Dublin, CA, USA). The detected bands were quantified using ImageJ 1.52a software (NIH, Bethesda, MD, USA).

### 2.9. Statistical Analysis

Statistical analyses were performed using SPSS software version 25.0 (SPSS, Chicago, IL, USA). Experiments were repeated at least three times, and statistical analyses were performed using Student’s *t*-test. Data are presented as the mean ± standard deviation (SD). A *p*-value < 0.05 was considered statistically significant.

## 3. Results

### 3.1. HPLC Analysis of A. japonicus Extract

**Figure 1:**
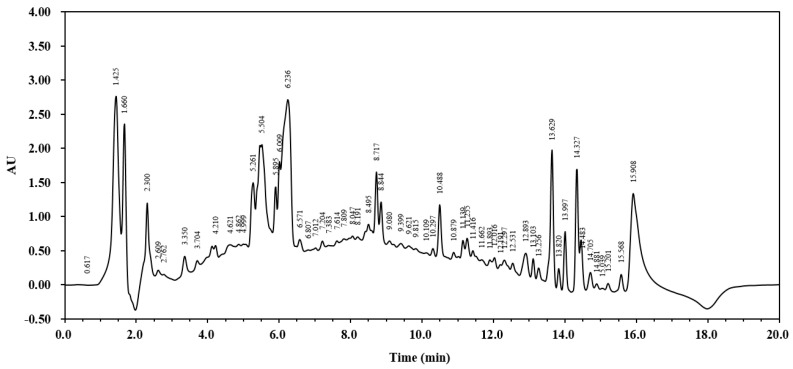
Typical preliminary HPLC chromatogram of a crude extract of *A. japonicus*. A 10 μL sample was injected into a reverse-phase column at a flow rate of 1 mL/min and monitored at 205 nm.

To demonstrate the complexity of the *A. japonicus* extract and justify the use of the crude extract over isolated compounds, a full-scan HPLC analysis was performed. The HPLC chromatogram of the crude extract of *A. japonicus* revealed a total of 60 peaks representing various bioactive compounds with different retention times. This complexity suggests potential synergistic effects among the compounds, which may enhance its bioactivity compared to that of its individual isolated components. The major peak, with an area value of 57,897,963, was observed at 15.908 min, while the lowest peak appeared at 0.617 min (Figure 1).

### 3.2. Impact of A. japonicus Extract on RAW 264.7 Cell Viability

**Figure 2:**
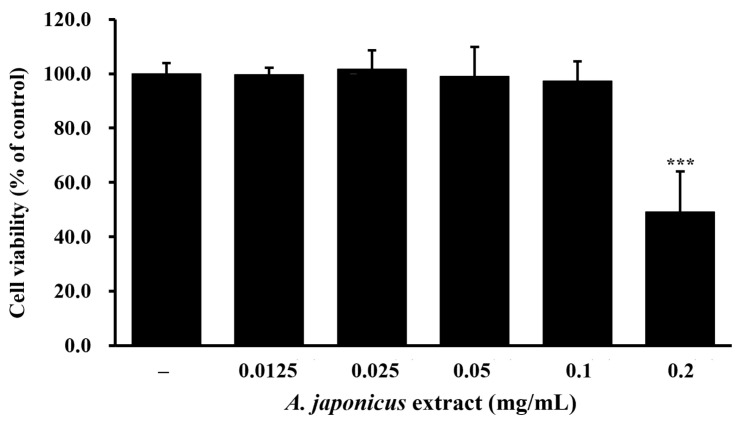
Effect of *Apostichopus japonicus* extract on the viability of RAW 264.7 cells. RAW 264.7 cells were treated with various concentrations of *A. japonicus* extract (0.0125–0.2 mg/mL) for 24 h, following which cell viability was determined by an MTS assay. Data are presented as the mean ± SD (*n* = 3). *** *p* < 0.001, compared with untreated cells.

First, we performed an MTS assay to determine the appropriate non-cytotoxic concentration range by confirming the cell viability of *A. japonicus* extract. RAW 264.7 cells were treated with various concentrations of *A. japonicus* extract (0.0125–0.2 mg/mL) for 24 h. No cytotoxic effects were observed at concentrations ranging from 0.0125 to 0.1 mg/mL (Figure 2). Therefore, *A. japonicus* extract did not affect RAW 264.7 cell viability at these concentrations and was analyzed for its anti-inflammatory effects within this non-cytotoxic range.

### 3.3. Effect of A. japonicus Extract on NO Production and iNOS Expression in P. gingivalis-Stimulated RAW 264.7 Cells

**Figure 3:**
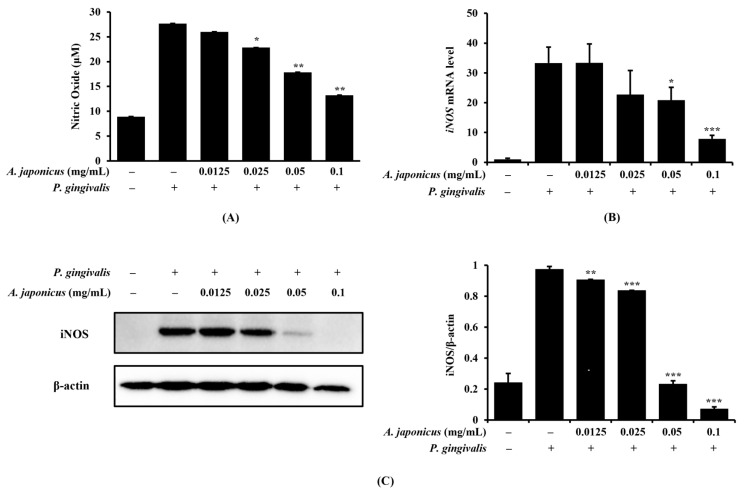
Inhibitory effect of *A. japonicus* extract on nitric oxide (NO) production and inducible nitric oxide synthase (iNOS) expression in *Porphyromonas gingivalis*-stimulated RAW 264.7 cells. RAW 264.7 cells were pretreated with various concentrations of *A. japonicus* extract (0.0125–0.1 mg/mL) for 2 h and then stimulated with *P. gingivalis* (1 × 10^7^ CFU/mL) for 24 h. (**A**) NO concentrations were measured using a Griess assay. The (**B**) mRNA and (**C**) protein levels of iNOS were determined by quantitative real-time PCR (qRT-PCR) and a Western blot, respectively. iNOS levels indicate changes in protein expression normalized to β-actin levels. Data are presented as the mean ± SD, in triplicate. * *p* < 0.05, ** *p* < 0.01, *** *p* < 0.001, compared to the *P. gingivalis*-stimulated cells.

Next, we investigated the effect of *A. japonicus* extract on NO production and iNOS expression in *P. gingivalis*-stimulated RAW 264.7 cells. While *P. gingivalis*-stimulated cells exhibited high levels of NO production, treatment with *A. japonicus* extract inhibited NO production (Figure 3A). iNOS, an enzyme that synthesizes NO, is expressed during inflammation. Using qRT-PCR and a Western blot, we confirmed that iNOS mRNA and protein levels were significantly increased in *P. gingivalis*-stimulated RAW 264.7 cells. However, treatment with *A. japonicus* extract decreased iNOS expression at both the mRNA and protein levels in a dose-dependent manner (Figure 3B,C). Therefore, *A. japonicus* extract inhibits NO production by downregulating iNOS expression.

### 3.4. Effect of A. japonicus Extract on Inhibition of Pro-Inflammatory Cytokine Expression in P. gingivalis-Stimulated RAW 264.7 Cells

**Figure 4:**
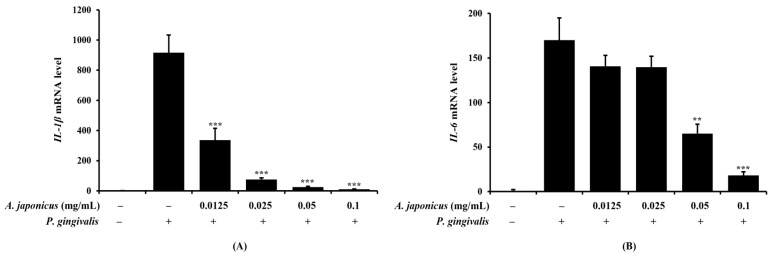
Effects of *A. japonicus* extract on interleukin (*IL*)-*1β* and *IL-6* production in *P. gingivalis*-stimulated RAW 264.7 cells. RAW 264.7 cells were pretreated with various concentrations of *A. japonicus* extract (0.0125–0.1 mg/mL) for 2 h and then stimulated with *P. gingivalis* (1 × 10^7^ CFU/mL) for 24 h. The mRNA levels of (**A**) *IL-1β* and (**B**) *IL-6* were measured by qRT-PCR. *IL-1β* and *IL-6* mRNA levels were normalized to *β-actin* mRNA levels. Data are presented as the mean ± SD, in triplicate. ** *p* < 0.01 and *** *p* < 0.001, compared with *P. gingivalis*-stimulated cells.

Pro-inflammatory cytokines play a crucial role in the inflammatory response, making the assessment of their expression essential to determining the anti-inflammatory effects of *A. japonicus* extract. We performed qRT-PCRs to analyze the mRNA levels of the key cytokines, *IL-1β* and *IL-6*, in *P. gingivalis*-stimulated RAW 264.7 cells pretreated with *A. japonicus* extract. The results showed that the mRNA expression of *IL-1β* and *IL-6* was reduced in a dose-dependent manner in the *A. japonicus* extract-treated groups compared to the *P. gingivalis*-only group (Figure 4A,B). These findings suggest that *A. japonicus* extract downregulates the mRNA expression of the pro-inflammatory cytokines induced by *P. gingivalis*.

### 3.5. Effect of A. japonicus Extract on the NF-κB Pathway in P. gingivalis-Stimulated RAW 264.7 Cells

**Figure 5:**
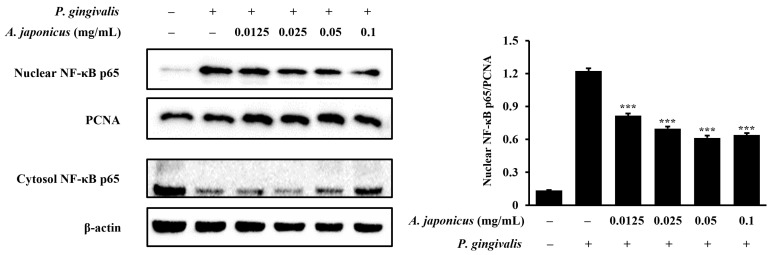
Effect of *A. japonicus* extract on nuclear factor kappa B (NF-κB) expression in *P. gingivalis*-stimulated RAW 264.7 cells. RAW 264.7 cells were pretreated with various concentrations of *A. japonicus* extract (0.0125–0.1 mg/mL) for 2 h and then stimulated with *P. gingivalis* (1 × 10^7^ CFU/mL) for 20 min. Subsequently, nuclear and cytoplasmic fractions were separated and subjected to Western blot analyses. NF-κB p65 levels were assessed in the nuclear and cytoplasmic fractions. Proliferating cell nuclear antigen (PCNA) and β-actin were used as loading controls for the nuclear and cytoplasmic extracts, respectively. The relative quantification of NF-κB p65 levels in the nuclear fraction was normalized to PCNA levels. Data are presented as the mean ± SD of triplicate experiments. *** *p* < 0.001, compared with *P. gingivalis*-stimulated cells.

NF-κB p65/p50 is a transcription factor that regulates the expression of inflammatory mediators [29]. Therefore, we examined whether *A. japonicus* extract affects the NF-κB signaling pathway, a key regulator of inflammatory responses. RAW 264.7 cells were pretreated with various concentrations of *A. japonicus* extract and then stimulated with *P. gingivalis*. A Western blot analysis was used to evaluate NF-κB p65 protein levels in the cytoplasm and nucleus. The results showed that the nuclear translocation of NF-κB p65 increased in *P. gingivalis*-stimulated cells compared to that in control cells, along with a reduction in cytoplasmic p65 levels. Treatment with *A. japonicus* extract reversed this effect, reducing nuclear NF-κB p65 expression while restoring its cytoplasmic levels (Figure 5). Therefore, *A. japonicus* extract inhibits the *P. gingivalis*-induced activation of the NF-κB pathway.

### 3.6. Effect of A. japonicus Extract on IκBα and IKKα/β Phosphorylation in P. gingivalis-Stimulated RAW 264.7 Cells

**Figure 6:**
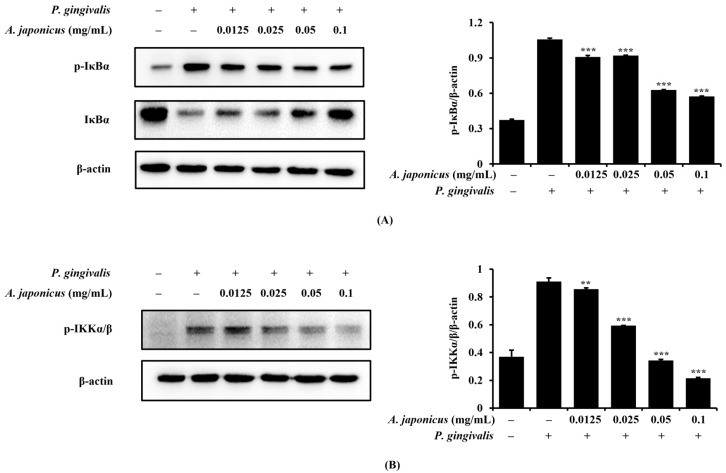
Effects of *A. japonicus* extract on NF-κB inhibitor α (IκBα) and IκB kinase (IKK)α/β phosphorylation in *P. gingivalis*-stimulated RAW 264.7 cells. RAW 264.7 cells were pretreated with various concentrations of *A. japonicus* extract (0.0125–0.1 mg/mL) for 2 h, followed by stimulation with *P. gingivalis* (1 × 10^7^ CFU/mL) for 10–15 min. Subsequently, cells were harvested and cell lysates were prepared for a Western blot analysis of the indicated proteins. (**A**) phosphorylated (p)-IκBα and IκBα levels in the cytoplasmic extracts. Relative quantification of IκBα phosphorylation normalized to β-actin levels. (**B**) p-IKKα/β levels in the cytoplasmic extracts. Relative quantification of IKKα/β phosphorylation normalized to β-actin levels. Data are expressed as the mean ± SD of triplicate experiments. ** *p* < 0.01 and *** *p* < 0.001, compared with *P. gingivalis*-stimulated cells.

To explore the mechanisms by which *A. japonicus* extract affects the NF-κB pathway, we investigated its impact on IκBα and IKKα/β phosphorylation, which are principal regulators of NF-κB activation. RAW 264.7 cells were pretreated with various concentrations of *A. japonicus* extract before stimulation with *P. gingivalis*. A Western blot analysis revealed that *P. gingivalis* significantly increased the phosphorylation of both IκBα and IKKα/β, which is critical for NF-κB activation. However, treatment with *A. japonicus* extract led to a reduction in the phosphorylation of IκBα and IKKα/β (Figure 6A,B). These results suggest that *A. japonicus* extract inhibits *P. gingivalis*-induced NF-κB activation by suppressing IκBα and IKKα/β phosphorylation, contributing to its anti-inflammatory effects.

### 3.7. Effect of A. japonicus Extract on MAPK (ERK, p38, and JNK) Activation in P. gingivalis-Stimulated RAW 264.7 Cells

**Figure 7:**
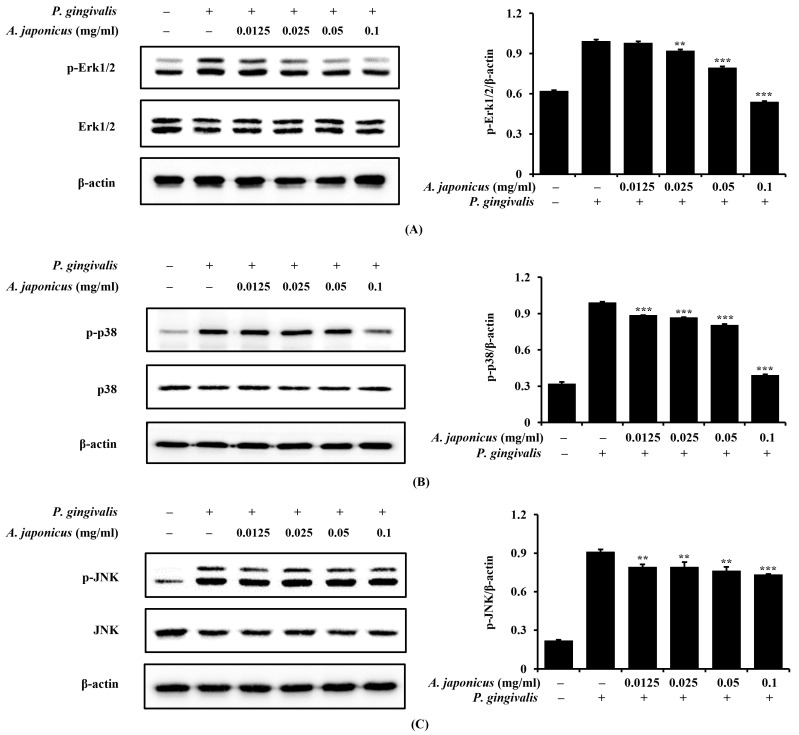
Effects of *A. japonicus* on the mitogen-activated protein kinase (MAPK) signaling pathway in *P. gingivalis*-stimulated RAW 264.7 cells. RAW 264.7 cells were pretreated with various concentrations of *A. japonicus* extract (0.0125–0.1 mg/mL) for 2 h and then stimulated with *P. gingivalis* (1 × 10^7^ CFU/mL) for 15 min. Cell lysates were analyzed using a Western blot analysis to assess the effect on the expression levels of (**A**) extracellular signal-regulated kinase (ERK), phosphorylated (p)-ERK, (**B**) p38, p-p38, and (**C**) c-Jun N-terminal kinase (JNK), p-JNK. All signals were normalized to β-actin levels, which served as an internal control. Data are expressed as the mean ± SD of triplicate experiments. ** *p* < 0.01 and *** *p* < 0.001, compared with the *P. gingivalis*-stimulated cells.

MAPK pathways, including ERK, p38, and JNK, are key mediators of inflammatory responses and contribute to NF-κB activation. To investigate the effect of the *A. japonicus* extract on MAPK activation, we pretreated RAW 264.7 cells with various concentrations of *A. japonicus* extract before stimulating them with *P. gingivalis*. A Western blot analysis showed that *P. gingivalis* induced the phosphorylation of ERK, p38, and JNK, whereas treatment with *A. japonicus* extract reduced the phosphorylation of these MAPKs (Figure 7A–C). These results suggest that *A. japonicus* extract inhibits *P. gingivalis*-induced MAPK activation, which may contribute to its overall inhibitory effect on NF-κB signaling.

## 4. Discussion

The inflammatory response is an essential defense mechanism against tissue damage, and the immune system is activated in response to an external pathogen or damage [30]. The inflammatory response plays an important role in localizing infection or damage and promoting recovery; however, excessive or chronic inflammation can have negative health effects. In particular, the long-term persistence of an inflammatory response in chronic inflammatory diseases such as periodontitis can worsen tissue damage and eventually lead to the destruction of teeth and surrounding tissues [31,32]. Currently, the treatment of periodontal disease mainly uses antibiotics and anti-inflammatory drugs, but the occurrence of antibiotic resistance during long-term use and side effects such as dry mouth have been pointed out as problems. Studies on anti-inflammatory compounds derived from natural products are attracting attention in an attempt to find safer and more effective alternatives while minimizing these side effects.

Notably, *A. japonicus* is a safe substance widely used as a food product and known for its diverse health benefits. Studies have demonstrated its anti-inflammatory, anti-allergic, antioxidant, and anti-melanogenic effects, which are attributed to various bioactive compounds such as saponins, polysaccharides, peptides, and polyphenols [22,25,26]. These compounds are believed to act synergistically, contributing to its therapeutic potential in the prevention and treatment of various diseases [33,34]. However, to date, studies on the effects of *A. japonicus* on the inflammatory response associated with periodontal diseases have not been reported. Therefore, the present study aimed to examine the anti-inflammatory effects of *A. japonicus* extract in RAW 264.7 cells stimulated with *P. gingivalis*.

Macrophages play an important role in the inflammatory response and, during the development of inflammation, macrophages, neutrophils, and other immune cells produce large amounts of NO as part of the immune response [35,36]. NO is a type of reactive oxygen species that is produced in L-arginine by NOS and plays an important role in inflammation induction [37]. In particular, iNOS is involved in the inflammatory response and is expressed in immune cells activated by immune stimulants, such as LPS. Although the expression of iNOS is not observed under normal conditions, iNOS induction through inflammatory stimulation can produce high concentrations of NO, mediating tissue damage, vasodilation, and nerve damage and thus exacerbating inflammation [38,39]. In this study, we evaluated the effect of *A. japonicus* extract on NO production in RAW 264.7 cells stimulated with *P. gingivalis*. The NO level was rarely detected in RAW 264.7 cells alone, but was remarkably increased after *P. gingivalis* stimulation. Interestingly, NO production was significantly inhibited at concentrations of 0.025–0.1 mg/mL, when *P. gingivalis*-stimulated cells were treated with *A. japonicus* extract. In addition, qRT-PCR and Western blot analyses confirmed that the mRNA and protein expressions of iNOS were significantly increased in RAW 264.7 cells stimulated with *P. gingivalis*. This expression was dose-dependently suppressed after treatment with *A. japonicus* extract. These results suggest that *A. japonicus* extract effectively inhibits *P. gingivalis*-induced iNOS expression, thereby reducing NO production.

iNOS expression is known to be regulated by NF-κB, an inflammatory transcription factor [40]. Under normal conditions, NF-κB is present in the cytoplasm and binds to IκBα; however, upon stimulation, the p65 subunit of NF-κB is phosphorylated and translocates to the nucleus, inducing the transcription of inflammatory genes [31,41]. The inhibition of NF-κB activity inhibits the phosphorylation of IκBα, thereby blocking the migration of NF-κB to the nucleus and inhibiting the inflammatory response. Considering that *A. japonicus* extract inhibited iNOS expression, we investigated its effect on the NF-κB pathway in *P. gingivalis*-stimulated RAW 264.7 cells. The *A. japonicus* extract inhibited the increase in NF-κB expression in the nucleus induced by *P. gingivalis* stimulation. It also inhibited the increase in p-IκBα levels and the decrease in IκBα levels in the cytoplasm, indicating its effective blocking of IκBα phosphorylation. In addition, the phosphorylation of IKKα/β induced by *P. gingivalis* was also inhibited following treatment with the *A. japonicus* extract. These results confirmed that the *A. japonicus* extract exhibited an anti-inflammatory effect through its inhibition of the NF-κB pathway.

The MAPK pathway plays an important regulatory role in the activation of NF-κB. ERK, p38, and JNK are signaling molecules of the MAPK pathway, each of which regulates the activity of NF-κB in response to various stimuli. ERK primarily promotes cell growth and survival, and p38 and JNK activate NF-κB in response to stress and inflammatory signals, leading to an inflammatory response [42,43]. NF-κB is a major transcriptional factor that regulates the expression of pro-inflammatory cytokines and other inflammation-inducing genes and, when activated through the MAPK pathway, the inflammatory response is amplified. Consequently, the MAPK pathway plays a key role in the regulation and amplification of the inflammatory response through NF-κB activation [8,44]. In this study, we confirmed that *A. japonicus* extract inhibited the MAPK signaling pathway in *P. gingivalis*-stimulated RAW 264.7 cells and found that the phosphorylation of ERK1/2, JNK, and p38 decreased in a dose-dependent manner upon treatment with the *A. japonicus* extract. This result suggests that the *A. japonicus* extract exhibited anti-inflammatory effects through the inhibition of the MAPK pathway and that the inhibition of the NF-κB pathway was also involved in this process.

Pro-inflammatory cytokines play an important role as a major mediator of the inflammatory response. Representative pro-inflammatory cytokines, including IL-1β, IL-6, and TNF-α, are secreted upon infection and tissue damage, promoting the migration of immune cells to the inflammatory site and amplifying the inflammatory response [16,45]. In this study, we found that in *P. gingivalis*-stimulated RAW 264.7 cells, *A. japonicus* extract inhibited the mRNA expression of *IL-1β* and *IL-6* in a dose-dependent manner. This suggests that *A. japonicus* extract can effectively regulate the inflammatory response by reducing the expression of inflammatory cytokines.

## 5. Conclusions

**Figure 8:**
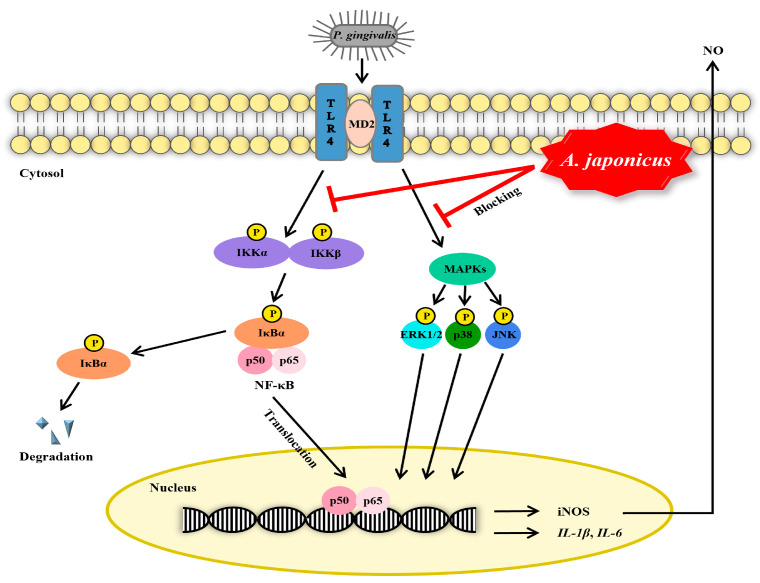
Schematic overview of the mechanisms underlying the anti-inflammatory effects of *Apostichopus japonicus* extract in *Porphyromonas gingivalis*-stimulated RAW 264.7 cells. IKK, IκB kinase; IκBα, NF-κB inhibitor α; NF-κB, nuclear factor kappa B; MAPK, mitogen-activated protein kinase; ERK, extracellular signal-regulated kinase; JNK, c-Jun N-terminal kinase; *IL*, interleukin; iNOS, inducible nitric oxide synthase; NO, nitric oxide.

According to our findings, *A. japonicus* extract inhibits the activation of the NF-κB and MAPK signaling pathways induced by *P. gingivalis* in RAW 264.7 cells, thereby inhibiting the synthesis of inflammatory mediators and pro-inflammatory cytokines (Figure 8). These results show that *A. japonicus* extract plays an important role in the regulation of the inflammatory response associated with periodontal disease. Therefore, considering the efficacy of *A. japonicus*, it can be used in the development of natural product-derived oral healthcare products.

## Data Availability

The original contributions presented in the study are included in the article; further inquiries can be directed to the corresponding author.
